# Supplementary material for: A new class of nonreciprocal spin waves on the edges of 2D antiferromagnetic honeycomb nanoribbons
Source: Sci Rep. 2019 Oct 23;9:15220. doi: 10.1038/s41598-019-51646-3 (PMC6811631; doi:10.1038/s41598-019-51646-3)
Supplement: Supplementary file 1 — Supplementary Information [file 41598_2019_51646_MOESM1_ESM.pdf]

Supplementary Information

A new class of nonreciprocal spin waves on the edges of 2D antiferromagnetic honeycomb nanoribbons

D. Ghader and A. Khater

Supplementary Note 1. Bulk equations of motion

Substituting equations 4 and 5 in the Bloch equations of motion

$$\partial_t \vec{M}^A = \lambda\, \vec{M}^A \times \vec{H}^A$$

yields 2 equations,

$$i\omega(A_x^+ e^{q\,y} + A_x^- e^{-q\,y}) = -3\gamma JM\left(A_y^+ e^{q\,y} + A_y^- e^{-q\,y}\right) - JM\left(B_y^+ e^{q\,y} f_{ex}^+ + B_y^- e^{-q\,y} f_{ex}^-\right)$$
$$+4iMDf_{DM}(A_x^+ e^{q\,y} + A_x^- e^{-q\,y}) \qquad \text{(S1)}$$

$$i\omega\left(A_y^+ e^{q\,y} + A_y^- e^{-q\,y}\right) = 3\gamma JM\,(A_x^+ e^{q\,y} + A_x^- e^{-q\,y}) + JM(B_x^+ e^{q\,y} f_{ex}^+ + B_x^- e^{-q\,y} f_{ex}^-)$$
$$+4iMDf_{DM}\left(A_y^+ e^{q\,y} + A_y^- e^{-q\,y}\right) \qquad \text{(S2)}$$

We next multiply S1 by $-i$ and add the result to S2 to get

$$e^{q\,y}\left[\left(-\Omega + \frac{4D}{J} f_{DM} + 3\gamma\right) A^+ + f_{ex}^+ B^+\right] + e^{-q\,y}\left[\left(-\Omega + \frac{4D}{J} f_{DM} + 3\gamma\right) A^- + f_{ex}^- B^-\right] = 0 \qquad \text{(S3)}$$

with $\Omega = \frac{\omega}{\lambda JM}$, $A^\pm = A_x^\pm + iA_y^\pm$, and $B^\pm = B_x^\pm + iB_y^\pm$.

Similar steps applied to the Bloch equation for a B-site, $\partial_t \vec{M}^B = \lambda\, \vec{M}^B \times \vec{H}^B$, yields

$$e^{q\,y}\left[\left(-\Omega + \frac{4D}{J} f_{DM} - 3\gamma\right) B^+ - f_{ex}^- A^+\right] + e^{-q\,y}\left[\left(-\Omega + \frac{4D}{J} f_{DM} - 3\gamma\right) B^- - f_{ex}^+ A^-\right] = 0 \qquad \text{(S4)}$$

Equations S3 and S4 hold for any y along the finite width of the nanoribbon. Consequently, the coefficients of $e^{\pm q\,y}$ should vanish which yields equations 6.

Supplementary Note 2. Edge equations

ZE nanoribbons

On the left edge of the nanoribbon ($y = d$),

$$\vec{H}_b^A - \vec{H}_e^A = -\gamma JM\hat{z} + \left\{-2i\phi D \sin\left(\frac{k_x a}{2}\right)\left[A_y^+ e^{q\left(d+\frac{\sqrt{3}a}{2}\right)} + A_y^- e^{-q\left(d+\frac{\sqrt{3}a}{2}\right)}\right] + J\phi\left[B_x^+ e^{q\left(d+\frac{a}{\sqrt{3}}\right)} + B_x^- e^{-q\left(d+\frac{a}{\sqrt{3}}\right)}\right]\right\}\hat{x}$$

$$+\left\{2i\phi D \sin\left(\frac{k_x a}{2}\right)\left[A_x^+ e^{q\left(d+\frac{\sqrt{3}a}{2}\right)} + A_x^- e^{-q\left(d+\frac{\sqrt{3}a}{2}\right)}\right] + J\phi\left[B_y^+ e^{q\left(d+\frac{a}{\sqrt{3}}\right)} + B_y^- e^{-q\left(d+\frac{a}{\sqrt{3}}\right)}\right]\right\}\hat{y}$$

The x and y components of the boundary equation $\vec{M}_e^A \times \left(\vec{H}_b^A - \vec{H}_e^A\right) = \vec{0}$ then yields the equations

$$\gamma\left(A_y^+ e^{qd} + A_y^- e^{-qd}\right) + \left[B_y^+ e^{q\left(d+\frac{a}{\sqrt{3}}\right)} + B_y^- e^{-q\left(d+\frac{a}{\sqrt{3}}\right)}\right] + i\frac{2D}{J}\sin\left(\frac{k_x a}{2}\right)\left[A_x^+ e^{q\left(d+\frac{\sqrt{3}a}{2}\right)} + A_x^- e^{-q\left(d+\frac{\sqrt{3}a}{2}\right)}\right] = 0 \qquad \text{(S5)}$$

$$\gamma\left(A_x^+ e^{qd} + A_x^- e^{-qd}\right) + \left[B_x^+ e^{q\left(d+\frac{a}{\sqrt{3}}\right)} + B_x^- e^{-q\left(d+\frac{a}{\sqrt{3}}\right)}\right] - i\frac{2D}{J}\sin\left(\frac{k_x a}{2}\right)\left[A_y^+ e^{q\left(d+\frac{\sqrt{3}a}{2}\right)} + A_y^- e^{-q\left(d+\frac{\sqrt{3}a}{2}\right)}\right] = 0 \qquad \text{(S6)}$$

Multiplying S5 by i then adding the result to S6 yields equation 10a.

We next consider the right edge at $y = -d$, with

$$\vec{H}_b^B - \vec{H}_e^B = \gamma JM\hat{z} + \left\{2i\phi D \sin\left(\frac{k_x a}{2}\right)\left[B_y^+ e^{-q\left(d+\frac{\sqrt{3}a}{2}\right)} + B_y^- e^{q\left(d+\frac{\sqrt{3}a}{2}\right)}\right] + J\phi\left[A_x^+ e^{-q\left(d+\frac{a}{\sqrt{3}}\right)} + A_x^- e^{q\left(d+\frac{a}{\sqrt{3}}\right)}\right]\right\}\hat{x}$$

$$+\left\{-2i\phi D \sin\left(\frac{k_x a}{2}\right)\left[B_x^+ e^{-q\left(d+\frac{\sqrt{3}a}{2}\right)} + B_x^- e^{q\left(d+\frac{\sqrt{3}a}{2}\right)}\right] + J\phi\left[A_y^+ e^{-q\left(d+\frac{a}{\sqrt{3}}\right)} + A_y^- e^{q\left(d+\frac{a}{\sqrt{3}}\right)}\right]\right\}\hat{y}$$

For the right boundary, the equation $\vec{M}_e^A \times \left(\vec{H}_b^A - \vec{H}_e^A\right) = \vec{0}$ gives

$$\gamma\left(B_y^+e^{-qd}+B_y^-e^{qd}\right)+\left[A_y^+e^{-q\left(d+\frac{a}{\sqrt{3}}\right)}+A_y^-e^{q\left(d+\frac{a}{\sqrt{3}}\right)}\right]$$
$$-i\,\frac{2D}{J}\sin\!\left(\frac{k_xa}{2}\right)\left[B_x^+e^{-q\left(d+\frac{\sqrt{3}a}{2}\right)}+B_x^-e^{q\left(d+\frac{\sqrt{3}a}{2}\right)}\right]=0 \qquad \text{(S7)}$$

$$\gamma(B_x^+e^{-qd}+B_x^-e^{qd})+\left[A_x^+e^{-q\left(d+\frac{a}{\sqrt{3}}\right)}+A_x^-e^{q\left(d+\frac{a}{\sqrt{3}}\right)}\right]$$
$$+i\,\frac{2D}{J}\sin\!\left(\frac{k_xa}{2}\right)\left[B_y^+e^{-q\left(d+\frac{\sqrt{3}a}{2}\right)}+B_y^-e^{q\left(d+\frac{\sqrt{3}a}{2}\right)}\right]=0 \qquad \text{(S8)}$$

Combining S7 and S8 then yields equation 10b.

BE nanoribbons

For BE nanoribbons,

$$\vec{H}_b^B-\vec{H}_e^B=2\gamma JM\hat{z}$$
$$+\left\{2i\phi D\sin\left(\frac{k_xa}{2}\right)\left[B_y^+e^{q\left(d+\frac{\sqrt{3}a}{2}\right)}+B_y^-e^{-q\left(d+\frac{\sqrt{3}a}{2}\right)}\right]+2J\phi\left[A_x^+e^{q\left(d+\frac{a}{\sqrt{3}}\right)}+A_x^-e^{-q\left(d+\frac{a}{\sqrt{3}}\right)}\right]\cos\left(\frac{k_xa}{2}\right)\right\}\hat{x}$$

$$+\left\{-2i\phi D\sin\left(\frac{k_xa}{2}\right)\left[B_x^+e^{q\left(d+\frac{\sqrt{3}a}{2}\right)}+B_x^-e^{-q\left(d+\frac{\sqrt{3}a}{2}\right)}\right]+2J\phi\left[A_y^+e^{q\left(d+\frac{a}{\sqrt{3}}\right)}+A_y^-e^{-q\left(d+\frac{a}{\sqrt{3}}\right)}\right]\cos\left(\frac{k_xa}{2}\right)\right\}\hat{y}$$

for the left edge, and

$$\vec{H}_b^A-\vec{H}_e^A=-2\gamma JM\hat{z}$$
$$+\left\{-2i\phi D\sin\left(\frac{k_xa}{2}\right)\left[A_y^+e^{-q\left(d+\frac{\sqrt{3}a}{2}\right)}+A_y^-e^{q\left(d+\frac{\sqrt{3}a}{2}\right)}\right]+2J\phi\left[B_x^+e^{-q\left(d+\frac{a}{\sqrt{3}}\right)}+B_x^-e^{q\left(d+\frac{a}{\sqrt{3}}\right)}\right]\cos\left(\frac{k_xa}{2}\right)\right\}\hat{x}$$

$$+\left\{2i\phi D\sin\left(\frac{k_xa}{2}\right)\left[A_x^+e^{-q\left(d+\frac{\sqrt{3}a}{2}\right)}+A_x^-e^{q\left(d+\frac{\sqrt{3}a}{2}\right)}\right]+2J\phi\left[B_y^+e^{-q\left(d+\frac{a}{\sqrt{3}}\right)}+B_y^-e^{q\left(d+\frac{a}{\sqrt{3}}\right)}\right]\cos\left(\frac{k_xa}{2}\right)\right\}\hat{y}$$

for the right edge.
The boundary equation $\vec{M}_e^A\times\left(\vec{H}_b^A-\vec{H}_e^A\right)=\vec{0}$ then yields,

$$\gamma\left(B_y^+ e^{qd} + B_y^- e^{-qd}\right) + \left[A_y^+ e^{q\left(d+\frac{a}{\sqrt{3}}\right)} + A_y^- e^{-q\left(d+\frac{a}{\sqrt{3}}\right)}\right]\cos\left(\frac{k_x a}{2}\right)$$

$$-i\frac{D}{J}\sin\left(\frac{k_x a}{2}\right)\left[B_x^+ e^{q\left(d+\frac{\sqrt{3}a}{2}\right)} + B_x^- e^{-q\left(d+\frac{\sqrt{3}a}{2}\right)}\right] = 0 \qquad \text{(S9)}$$

$$\gamma(B_x^+ e^{qd} + B_x^- e^{-qd}) + \left[A_x^+ e^{q\left(d+\frac{a}{\sqrt{3}}\right)} + A_x^- e^{-q\left(d+\frac{a}{\sqrt{3}}\right)}\right]\cos\left(\frac{k_x a}{2}\right)$$

$$+i\frac{D}{J}\sin\left(\frac{k_x a}{2}\right)\left[B_y^+ e^{q\left(d+\frac{\sqrt{3}a}{2}\right)} + B_y^- e^{-q\left(d+\frac{\sqrt{3}a}{2}\right)}\right] = 0 \qquad \text{(S10)}$$

for the left edge, and

$$\gamma\left(A_y^+ e^{-qd} + A_y^- e^{qd}\right) + \left[B_y^+ e^{-q\left(d+\frac{a}{\sqrt{3}}\right)} + B_y^- e^{q\left(d+\frac{a}{\sqrt{3}}\right)}\right]\cos\left(\frac{k_x a}{2}\right)$$

$$+i\frac{D}{J}\sin\left(\frac{k_x a}{2}\right)\left[A_x^+ e^{-q\left(d+\frac{\sqrt{3}a}{2}\right)} + A_x^- e^{q\left(d+\frac{\sqrt{3}a}{2}\right)}\right] = 0 \qquad \text{(S11)}$$

$$\gamma(A_x^+ e^{-qd} + A_x^- e^{qd}) + \left[B_x^+ e^{-q\left(d+\frac{a}{\sqrt{3}}\right)} + B_x^- e^{q\left(d+\frac{a}{\sqrt{3}}\right)}\right]\cos\left(\frac{k_x a}{2}\right)$$

$$-i\frac{D}{J}\sin\left(\frac{k_x a}{2}\right)\left[A_y^+ e^{-q\left(d+\frac{\sqrt{3}a}{2}\right)} + A_y^- e^{q\left(d+\frac{\sqrt{3}a}{2}\right)}\right] = 0 \qquad \text{(S12)}$$

for the right edge.

Combining equations S9 and S10 (respectively S11 and S12) yields equation 11a (respectively 11b).
